# High‐frequency assessment methods to detect subtle change in cognitive worsening

**DOI:** 10.1002/alz.094706

**Published:** 2025-01-09

**Authors:** Andrew J. Aschenbrenner

**Affiliations:** ^1^ Washington University School of Medicine in St. Louis, St. Louis, MO USA

## Abstract

**Background:**

Several trials using BACE inhibitors have stopped for futility, and in some cases, evidence of progressive cognitive worsening, particularly early in treatment. As new trials are launched, evaluations of acute cognitive decline need to be made at the individual level, so that treatment can be stopped or adjusted if there is evidence of sufficient cognitive decline. To this end, it is essential to adopt assessment methodologies that can monitor *cognitive safety* and provide benchmarks against which to compare acute change. A cognitive safety monitoring system in this context must be psychometrically reliable, sensitive to change, and tolerable by participants. We examine the suitability of a high‐frequency, remote cognitive assessment study (Ambulatory Research in Cognition [ARC]) for this purpose. Its sensitivity can be established by administering controlled cognitive challenges, such as 24‐hour sleep restriction. Consistent cognitive change that exceeds this magnitude can serve as an early warning of adverse cognitive effects.

**Method:**

To date, ARC has enrolled and continuously followed ∼350 healthy and very mildly impaired older adults using brief cognitive tests across three domains. Tests were administered four times daily for one week and repeated at six‐month intervals. Adherence and reliability of these tests have previously been shown to be excellent. We compared performance on the cognitive tests at the beginning and end of the day as a proxy for sleep restriction.

**Results:**

Average response time at the start of the day was 3.4 seconds (SD = 1.12) and at the end of the day response time was 3.6 seconds (SD = 1.1). This difference was marginally significant based on a paired samples t‐test (p = 0.07, Cohen’s d = 0.19).

**Conclusion:**

Remote, high‐frequency cognitive assessment is feasible and tolerable by healthy and impaired participants. More importantly, this assessment methodology affords the ability to measure variability and can detect even subtle cognitive deficits associated with sleep restriction. In our presentation, we will expand on our methods for developing the construct of cognitive safety and provide additional details on how this system could be used in upcoming BACE inhibitor secondary prevention trials.

